# Thermosensitive Sprayable Lidocaine–Allantoin Hydrogel: Optimization and In Vitro Evaluation for Wound Healing

**DOI:** 10.3390/pharmaceutics17121607

**Published:** 2025-12-14

**Authors:** Muhammet Davut Arpa, Sevde Nur Biltekin Kaleli

**Affiliations:** 1 Department of Pharmaceutical Technology, School of Pharmacy, Istanbul Medipol University, 34815 Istanbul, Türkiye; 2 Department of Pharmaceutical Microbiology, School of Pharmacy, Istanbul Medipol University, 34815 Istanbul, Türkiye; snbiltekin@medipol.edu.tr

**Keywords:** thermosensitive hydrogel, spray formulation, in situ gel, stimuli-responsive hydrogel, wound healing, lidocaine, allantoin, response surface methodology, scratch assay

## Abstract

**Background/Objectives:** Wound healing requires simultaneous pain control, inflammation management, infection prevention, and tissue regeneration. This study aimed to develop and evaluate in vitro a non-contact thermosensitive spray hydrogel combining lidocaine for rapid analgesia and allantoin for tissue repair. **Methods**: The effects of chitosan and Poloxamer 407 on viscosity, spray diameter, and bioadhesion ability of hydrogels were optimized using response surface methodology. Lead formulations (S1 and S2) were selected via a desirability function within the software. The pH, gelation temperature (T_G_), rheological behavior, sprayability, bioadhesion, and lidocaine release using the dialysis bag method were assessed. The in vitro cytotoxicity, anti-inflammatory activity (TNF-α), and cell migration (scratch assay) of the formulations were investigated. **Results**: The viscosity values (42.7–58.7 mPa·s) indicated suitability for spraying at room temperature. T_G_ was 28.7 ± 0.6 °C (S1) and 29.3 ± 0.3 °C (S2), enabling rapid sol–gel transition at skin temperature. The lidocaine release reached 95–100% within 120 min. S2 exhibited lower viscosity and wider spray diameter, improving applicability on larger wound areas. In vitro cytotoxicity, scratch assay, and inflammatory marker analyses demonstrated that the optimized sprayable hydrogels exhibited a biocompatible and cell-healing profile. **Conclusions**: The developed thermosensitive spray hydrogel enables the combined delivery of lidocaine and allantoin, rapid gelation at body temperature, and touch-free administration. Its suitable viscosity and sprayability, and fast lidocaine release profile indicate high patient compliance and a significant advantage over conventional cream/ointment formulations, particularly regarding painless application, reduced contamination risk, enhanced therapeutic potential, and confirmed in vitro biocompatibility with supportive effects on keratinocyte behavior.

## 1. Introduction

Wounds occur as a result of structural deterioration, ranging from superficial epithelial damage to deep tissue loss, and chronic wounds in particular are a serious health condition that affects 1% to 2% of people in developed nations [1]. Wound healing is a complex biological process involving the phases of hemostasis, inflammation, proliferation, and remodeling, which collectively aim to restore tissue integrity and function [2,3]. In order for this process to proceed properly, infection at the wound site should be controlled, an appropriate moisture balance maintained, and cellular regeneration supported [4]. Topical drug delivery offers a safer and more effective approach to achieving these goals compared to systemic treatments [5]. However, traditional topical formulations are challenging to use due to their limited applicability on irregular or uneven wound surfaces and their short duration of action, which limits wound-healing effectiveness [6]. Moreover, the amount applied, especially in semi-solid topical dosage forms, is often determined by the patient’s judgment, leading to individual dosage variability and poor treatment compliance [7]. To overcome these disadvantages, sprayable thermosensitive gel systems have gained prominence in recent years [8,9,10]. These systems can be applied in liquid form at low temperatures and undergo gelation at body temperature, adhering homogeneously to the wound surface [8]. This allows for a metered, uniformly distributed, and contactless application, improving patient comfort and making them of great interest for the treatment of painful wounds [11]. Furthermore, the elevated water content of spray hydrogels contributes to maintaining tissue homeostasis within the wound microenvironment [12].

Poloxamers are amphiphilic triblock copolymers consisting of poly(ethylene oxide) and poly(propylene oxide) segments, and they exhibit reversible gelation with temperature. Poloxamer 407 (P407), due to its low toxicity, high solubilization capacity, and biomolecular compatibility, undergoes gelation at body temperature, enabling the sustained release of the active compound [13]. It has also been reported to function as an efficient delivery platform in wound-healing applications [14]. However, the poor mechanical strength and low bioadhesive properties of pure poloxamer gels, along with their short residence time, may limit their formulation effectiveness [15]. Therefore, their combination with biocompatible, biodegradable, and bioadhesive natural polymers such as chitosan has become a widely adopted strategy [16,17,18]. In addition, chitosan, which is commonly used in wound healing due to its non-antigenic and antimicrobial properties, supports healing by accelerating cell proliferation and wound contraction [19]. Consequently, it has been employed in various wound-treatment formulations such as hydrogels, films, and sponges [19,20,21].

The thermosensitive spray-gel system developed in this study is composed of P407 and chitosan and incorporates two therapeutic agents: lidocaine hydrochloride and allantoin. Lidocaine is a local anesthetic that reversibly blocks nerve conduction by inhibiting voltage-gated sodium channels, thereby rapidly relieving pain at the wound site. Furthermore, in some cases, it can also exhibit analgesic activity independent of its local anesthetic effect. In addition, it suppresses the activation of inflammatory cells and reduces the release of pro-inflammatory mediators such as TNF-α and IL-6, thus providing a local anti-inflammatory effect [22]. Allantoin is a plant-derived compound found in species such as *Aloe vera* and *Calendula officinalis*. Its biocompatible structure and anti-inflammatory and anti-nociceptive effects promote tissue regeneration and wound healing. Moreover, studies have demonstrated that allantoin exhibits neuroregenerative, antibacterial, and antifungal activities [23]. These versatile biological effects of allantoin have been linked to its ability to promote cell proliferation and epithelialization. It is thought to accelerate new tissue formation by facilitating the removal of necrotic tissue at the cellular level. Furthermore, its anti-irritant, soothing, and mild analgesic effects contribute to the healing process by reducing discomfort and inflammation at the wound site [24]. These advantages have made allantoin a widely used ingredient in topical wound-care formulations [25,26,27].

To the best of our knowledge, no hydrogel-based system incorporating the combined use of lidocaine and allantoin for wound management has been reported in the literature. However, Yaşayan et al. (2021) [28] developed a film formulation containing both active ingredients. Additionally, several marketed topical products containing lidocaine and allantoin, either together or in combination with other active ingredients, are available in Europe and the USA [29,30,31]. While these examples support the clinical applicability and safety of the combination, they also indicate that current formulations, due to their semi-solid nature, have limitations in terms of dose control, ease of application, and surface uniformity. In this context, the thermosensitive spray-gel system developed in our study aims to enable this combination to be applied to the wound surface in a non-contact, metered, and homogeneously distributed manner.

The combined use of these two components aims to provide a synergistic effect in wound treatment, regarding both symptomatic relief (pain control) and causal healing (supporting tissue regeneration). This approach reduces the likelihood that patients will avoid dressing changes or topical applications due to pain, thereby improving treatment compliance and overall wound care effectiveness. Furthermore, combining lidocaine’s analgesic effect with allantoin’s regenerative effect in a single formulation minimizes the need for multiple products and reduces the risk of contamination.

The formulations were developed and optimized using response surface methodology (RSM). This statistical approach enables multivariate analysis to evaluate the effects of P407 and chitosan concentrations on critical formulation quality attributes—viscosity, sprayability, and bioadhesion. The obtained data were optimized using the desirability function to identify the lead formulations, which were then characterized in detail for their physicochemical properties, in vitro release profiles, and biological activities. The developed hydrogel was designed to serve as an innovative wound-treatment formulation, offering combined drug delivery, high bioadhesion, suitable viscosity, and efficient sprayability, thereby providing an alternative to conventional topical formulations.

## 2. Materials and Methods

### 2.1. Materials

Lidocaine hydrochloride (Suvar Kimya, İstanbul, Türkiye), allantoin (Doğa İlaç, İstanbul, Türkiye), P407 (BASF, Ludwigshafen, Germany), chitosan (BBI Life Sciences, Shanghai, China), 3-(4,5-dimethylthiazol-2-yl)-2,5-diphenyltetrazolium bromide (MTT) and glutamic acid (Sigma-Aldrich, St. Louis, MO, USA), and acetonitrile (Merck, Darmstadt, Germany) were used. P407 was kindly provided as a gift by BASF. Reagents used for cell culture were purchased from Gibco (Paisley, UK). The TNF-α assay kit was purchased from Elabscience (Houston, TX, USA). The human keratinocyte cell line (HaCaT) was obtained from the American Type Culture Collection (ATCC, Rockville, MD, USA). All chemicals were of pharmaceutical grade and used without additional purification.

### 2.2. Experimental Design

Optimization of chitosan- and P407-based spray-gel formulations was performed using Design-Expert software (Version 22.0.3, Stat-Ease Inc., Minneapolis, MN, USA). The optimization study was conducted within the framework of the Quality by Design (QbD) approach using RSM. P407 and chitosan concentrations were selected as independent variables, with the minimum and maximum levels set at 14–18% for P407 and 0.1–0.5% for chitosan (Table 1). The factor ranges for P407 were determined based on a previous in situ gel study [10], while the chitosan range was established from viscosity values obtained in preliminary trials.

The selected factor levels were entered into the software to generate an experimental design consisting of 21 runs (Table 2). Spray pattern diameter, work of adhesion, and viscosity were defined as response parameters to evaluate the effect of formulation variables. Response surface plots were constructed using the experimental results, and the optimal formulation was identified from the model-predicted target responses.

### 2.3. Preparation of Spray-Gel Formulations

**Figure 1:**
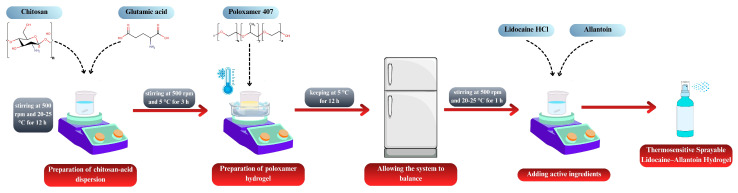
Schematic illustration of the preparation of thermosensitive sprayable lidocaine–allantoin hydrogel.

The formulations generated according to the RSM design were prepared as follows. Chitosan and glutamic acid were dispersed in distilled water at a 1:1 molar ratio [32]. The dispersion was stirred at 500 rpm for 12 h at room temperature, protected from light, using a magnetic stirrer (MR Hei-Standard, Heidolph Instruments, Schwabach, Germany). P407 was then incorporated into the chitosan dispersion using the cold method [33]. All procedures were carried out at 5 ± 2 °C, and P407 was slowly added to the pre-cooled dispersion. To prevent gelation, the temperature was maintained constant, and homogeneity was ensured throughout the mixing process. The mixture was stirred at 500 rpm for 3 h, and the final dispersions were stored in a refrigerator overnight to allow for complete equilibration of the system. Subsequently, lidocaine hydrochloride (5%, *w*/*w*) and allantoin (0.5%, *w*/*w*) were incorporated into the equilibrated gels and mixed for 1 h at room temperature to obtain a homogeneous drug-loaded hydrogel. Figure 1 illustrates the process for preparing the spray-gel formulations.

### 2.4. Characterization of Spray-Gel Formulations

#### 2.4.1. Viscosity

Viscosity values of the formulations were determined using a Brookfield DV2T RV viscometer (Brookfield Engineering Laboratories, Middleboro, MA, USA). The formulations were kept in the refrigerator and carefully transferred to the metal tubes before measurement, and an external thermometer was used for each measurement to ensure accurate temperature adjustment. Measurements were started when the temperature reached the target value. Viscosity analyses were carried out using an SC21-type spindle at a constant speed of 50 rpm, at 5 ± 1 °C. Each measurement was performed three times, and data are presented as the mean ± standard deviation (SD).

#### 2.4.2. Sprayability

The sprayability of the formulations was assessed according to previously reported procedures [10], with several experimental conditions refined to suit the present study. A TA.XTplusC texture analyzer (Stable Micro Systems, Haslemere, Surrey, UK) equipped with a 5 kg load cell was utilized to apply a consistent force during each spraying operation. Prior to measurement, a specific amount of red dye was added to the formulations, and the mixtures were stirred at 5 °C for 15 min until completely dispersed. The samples were then transferred into polyethylene spray bottles (20 mL) and stored in a refrigerator (5 °C) until use. To maintain a constant temperature during the experiment, the spray bottles were placed in a cold water bath (5 ± 2 °C). A flat-surface probe (SPS P/35, Ø = 35 mm) was used to actuate the sprays. The probe was moved downward at 30 mm/s with the device’s “down” function, and spraying was performed. A sheet of cellulose filter paper, positioned vertically at a distance of 20 cm from the bottle, served as the target surface. The spray pattern diameter was measured at two perpendicular points, and the mean value was calculated. All experiments were performed in triplicate. After spraying, the presence of any gel residue or leakage around the nozzle was visually inspected.

#### 2.4.3. In Vitro Bioadhesion Tests

To compare the bioadhesive properties of the spray hydrogels, in vitro bioadhesion tests were performed using the texture analyzer. The methods reported in the literature were employed [10,34], with minor modifications to suit the purpose of the study. A cellulose mixture–hydrophilic filter paper (0.45 µm, Sartorius, Göttingen, Germany) was used as the model membrane. The membrane was affixed to the lower surface of a Perspex SNSP/10 cylindrical probe (Ø = 10 mm) using double-sided adhesive tape and trimmed into a circular shape matching the probe area. A beaker containing 1 g of gel was weighed and secured to the heating platform with double-sided tape. The pre-test, test, and post-test speeds were adjusted to 0.5, 0.5, and 0.1 mm/s, respectively. The contact time between the gel and the membrane was set to 60 s. To maintain the gel surface temperature at 32 ± 1 °C, the heating plate (HSC-19T, Joan Lab, Huzhou, China) was adjusted to 34 °C. All experiments were conducted in four replicates. The maximum detachment force recorded during membrane-hydrogel separation was defined as the adhesive force. Additionally, using the instrument software, the work of adhesion was determined from the area under the force–distance curve.

### 2.5. Optimization and Preparation of Lead Formulations

Optimization of the formulations was carried out using the RSM approach based on a quadratic model. After all experimental data of the DoE-designed formulations (F1–F9) were entered into the software, the optimal composition for the drug-loaded formulations was determined based on the obtained responses (spray diameter, bioadhesion, and viscosity). Two different optimization strategies were applied. In the first approach, the spray diameter and bioadhesion were targeted for maximization, while viscosity was minimized. In this setup, the concentration of P407 was kept at a minimum, whereas the chitosan concentration was fixed at its maximum. In the second optimization approach, similar objectives were maintained; however, the chitosan concentration was restricted to be “in range”. The optimized drug-loaded formulations obtained from these two strategies were designated as S1 and S2, respectively. These lead spray gels (S1 and S2) were prepared according to the procedure described previously.

### 2.6. Characterization of Lead Hydrogels

#### 2.6.1. Viscosity, Sprayability, and In Vitro Bioadhesion

Viscosity (at 5 °C), sprayability, and bioadhesion measurements were performed using the same procedure as described above.

#### 2.6.2. pH

pH measurements were conducted using a calibrated pH meter (HI991001, Hanna Instruments, Woonsocket, RI, USA). Before measurement, the probe was immersed in the gel, and readings were taken after the value had stabilized. For each formulation, measurements were performed at two different points from three independent samples, and the mean pH value was calculated.

#### 2.6.3. Gelation Temperature (T_G_)

The T_G_ of the spray gels was investigated using the classical magnetic stirrer method reported in the literature and was further supported by temperature-dependent viscosity measurements. Initially, T_G_ was measured according to the method described by Aksu et al. (2019) [35], with minor modifications. Briefly, 10 ± 0.1 g of hydrogel stored at 5–8 °C was accurately weighed and transferred into a beaker. The beaker was placed on a magnetic hot plate stirrer (RT 15, IKA, Staufen, Germany), and the stirring speed was maintained at 100 rpm. The temperature was monitored using a digital thermometer immersed in the beaker and increased at a controlled rate of 0.5 °C/min. The T_G_ was defined as the temperature at which the magnetic stir bar stopped rotating completely or began rotating irregularly. To verify gel formation not only macroscopically but also rheologically, temperature-dependent viscosity changes were evaluated using the Brookfield DV2T viscometer (SC21 spindle, 50 rpm). The measurements were taken starting at 5 °C, increasing the temperature, and the test was terminated when the viscosity reached 1000 mPa·s (the upper measurement limit of the device). Additionally, to evaluate whether the active ingredients affect the T_G_, the classical T_G_ measurement described above was further performed on the drug-free versions of the lead formulations (S1_blank_ and S2_blank_).

#### 2.6.4. Content Uniformity

Content uniformity analyses were performed using a UV–Visible spectrophotometer (UV-6100PC, Mapada, Shanghai, China). An accurately weighed 500 ± 5 mg portion of each gel formulation was transferred to a volumetric flask (100 mL). The samples were homogenized by dispersing them in an acetonitrile–water mixture (1:1, *v*/*v*), filtered into quartz cuvettes, and measured against a blank solution. Stock solutions of both active ingredients were prepared for quantitative analysis. For lidocaine hydrochloride, a stock solution (1 mg/mL) was prepared by dissolving 100 mg of the compound in 100 mL ACN: water (1:1, *v*/*v*). From this stock, a series of standard solutions was prepared at concentrations of 10, 25, 50, 100, 150, 200, and 250 µg/mL. Similarly, for allantoin, a stock solution (1 mg/mL) was prepared in the same solvent system and diluted to obtain an intermediate stock solution of 100 µg/mL. From this, diluted samples were prepared at concentrations of 1, 2.5, 5, 10, 15, 20, and 25 µg/mL. The maximum absorbance wavelengths (λ_max_) were determined individually for each compound before analysis and were identified as 263 nm for lidocaine hydrochloride and 211 nm for allantoin. All solutions were prepared in triplicate, and absorbance values were recorded using the UV spectrophotometer under identical conditions.

### 2.7. Drug Release Studies

Drug release profiles were determined using the dialysis bag method. An accurately weighed 1.00 ± 0.01 g of each gel formulation was transferred into dialysis bags with a molecular weight cut-off (MWCO) of 12,000–14,000 Da (Spectrum Laboratories, Rancho Dominguez, CA, USA), and the ends were tightly sealed. The studies were conducted in glass beakers containing 200 mL of pH 7.4 phosphate buffer. The system was maintained on the magnetic hot plate stirrer at 32 ± 1 °C and a stirring speed of 50 rpm [10,35]. As a control, an aqueous solution containing the same concentration of active ingredients, 5% (*w*/*w*) lidocaine hydrochloride and 0.5% (*w*/*w*) allantoin, was prepared and tested under identical conditions. Samples (3 mL) were withdrawn at predetermined intervals (15, 30, 60, 90, 120, 180, and 240 min) and immediately replaced with an equivalent amount of fresh buffer to preserve sink conditions. All experiments were performed in four independent replicates. The collected samples were filtered and analyzed using the UV–Visible spectrophotometer. The amount of lidocaine hydrochloride released into the diffusion medium was quantified using absorbance measurements at 263 nm. Furthermore, the release profiles obtained were evaluated for similarity using the similarity factor (f_2_).

### 2.8. Cell Culture

Human keratinocyte cell line (HaCaT) was used for in vitro cytotoxicity and scratch experiments. The cells were cultivated in Dulbecco’s Modified Eagle Medium (DMEM, high glucose) supplemented with a standard antibiotic–antimycotic cocktail that contained amphotericin B (0.25 µg/mL), streptomycin (100 µg/mL), and penicillin (100 U/mL) along with 10% (*v*/*v*) heat-inactivated fetal bovine serum and 2 mM L-glutamine. The cultures were kept in a humidified incubator with 5% CO_2_ at 37 °C. The medium was refreshed every 2–3 days, and subculturing was performed upon reaching approximately 80–90% confluency to ensure exponential cell growth and viability.

#### Cytotoxicity Analyses

The potential cytotoxic effects of the formulations on HaCaT cells were evaluated by the MTT assay, which demonstrates mitochondrial metabolic activity as an indicator of cell viability. Before treatment, HaCaT cells were allowed to adhere for 24 h after being seeded into 96-well plates at a density of 1 × 10^5^ cells per well. After incubation with the test formulations for the designated period, the culture medium was replaced with 30 µL of MTT reagent (0.5 mg/mL in sterile PBS), followed by a 4 h incubation at 37 °C to allow for the formation of insoluble formazan crystals. These crystals were subsequently dissolved in 150 µL of isopropanol, and the absorbance was quantified at 570 nm using a microplate reader (SpectraMax i3, Molecular Devices, San Jose, CA, USA). All conditions were tested in triplicate and replicated in three independent experiments [36].

### 2.9. Wound-Healing Activity of Formulations on HaCaT Keratinocytes

A cell culture–based wound healing model was employed to evaluate the effects of formulations (S1, S2, S1_blank_, S2_blank_, S1_blank&chi-free_, S1_lido_, S2_lido_, S1_all_, and S2_all_) on cellular migration and proliferation. S1 and S2 were both allantoin- and lidocaine hydrochloride-loaded formulations, while S1_blank_ and S2_blank_ were drug-free formulations. S1_blank&chi-free_ was a formulation containing only P407, without chitosan. S1_lido_ and S2_lido_ were lidocaine hydrochloride-loaded formulations, while S1_all_ and S2_all_ were allantoin-loaded formulations. Briefly, cells were seeded into 24-well culture plates at a density of 1.5 × 10^5^ cells/well in 0.4 mL of growth medium and incubated for 24 h until a confluent monolayer was achieved. Subsequently, a linear scratch was made across each well using a 200 µL sterile pipette tip, creating a wound area. This scratch disrupted only the cell layer; no hydrogel formulation was applied on the surface at the first stage. The cell debris generated within the scratched area was gently washed twice with phosphate-buffered saline (PBS) to remove debris and then replaced with 0.5 mL of high-glucose DMEM supplemented with 10% fetal bovine serum, containing equal concentrations (1 mg/mL) of the hydrogel formulations. The microscopic images of the wounded areas were captured at 0 and 24 h post-scratch under 10× magnification using an AxioCam microscope (Carl Zeiss, Oberkochen, Germany) [37].

### 2.10. Effect of Formulations on TNF-α Expression Levels

To determine the anti-inflammatory potential of the formulations, TNF-α concentrations in the cell culture supernatants were quantified using commercial ELISA kits (Elabscience, Houston, TX, USA; EIAab, Wuhan, China) according to the manufacturers’ instructions. Cells were seeded into 96-well plates at a density of 1 × 10^5^ cells/mL and incubated for 24 h at 37 °C under a humidified 5% CO_2_ atmosphere to allow cell attachment and stabilization. Inflammatory responses were induced by exposure to lipopolysaccharide (LPS), followed by treatment with the test formulations at concentrations previously confirmed to be non-cytotoxic. After the incubation period, the cell culture supernatants were harvested by gentle centrifugation (2000× *g*, 3–30 K centrifuge, Sigma, Osterode am Harz, Germany) to eliminate cellular debris. The clarified supernatants were subsequently analyzed for TNF-α level via ELISA, and the optical density was recorded at 450 nm using the microplate reader. Each condition was tested in triplicate, and the entire experiment was repeated at least three times independently to ensure reproducibility and reliability of the data [36].

### 2.11. Statistical Analysis

All experimental data are expressed as mean ± SD. One-way analysis of variance (ANOVA) was used to assess differences between groups, and multiple comparisons were performed using Dunnett’s or Tukey’s post hoc tests. Data analysis was performed using Prism 9.0 (GraphPad Software, San Diego, CA, USA). A *p*-value of less than 0.05 was considered statistically significant.

## 3. Results and Discussion

RSM, a mathematical and statistical approach, was employed to optimize the formulation design by minimizing the number of independent variables [38,39]. Nine formulations (F1–F9) were constructed according to the RSM design. The repeated center points in the experimental matrix corresponded to formulations with identical compositions, and the mean values of these replicates were used for statistical analysis. The key response parameters (spray pattern diameter, viscosity, and work of bioadhesion) were determined for all formulations. Optimization based on a quadratic response surface model was performed using data obtained from drug-loaded formulations, representing the final therapeutic system.

Model fit parameters were analyzed to evaluate the statistical significance and predictive reliability of the generated mathematical models for viscosity, spray diameter, and adhesion strength (Table 3). For all responses, the selected quadratic model showed strong correlations with the experimental data, as indicated by high coefficients of determination (R^2^ > 0.96), adjusted R^2^ (>0.94), and predicted R^2^ (>0.92). Furthermore, Adeq Precision values exceeding 4 (24.26–31.60) demonstrated a sufficient signal-to-noise ratio, confirming that the models were statistically robust and reliable within the defined design space. These findings indicate that RSM-based models can accurately predict response outcomes that depend on formulation parameters.

The ANOVA results (Table 4) demonstrated that the quadratic model was statistically significant for all responses (*p* < 0.0001). The model F-values were 101.29 for viscosity, 76.80 for spray diameter, and 98.37 for bioadhesion, confirming that the variability in these responses is attributable to formulation factors rather than random error. Both main factors, chitosan (A) and P407 (B), exhibited significant effects on all responses. Additionally, for certain responses, the interaction term (AB) and quadratic terms (A^2^ and B^2^) also contributed significantly to the model. The high model F-values, low residual error, and non-significant Lack of Fit values (*p* > 0.05) collectively indicate that the selected quadratic model is appropriate, reliable, and accurately represents the experimental data.

### 3.1. Characterization of Spray-Gel Formulations

#### 3.1.1. Viscosity

**Figure 2:**
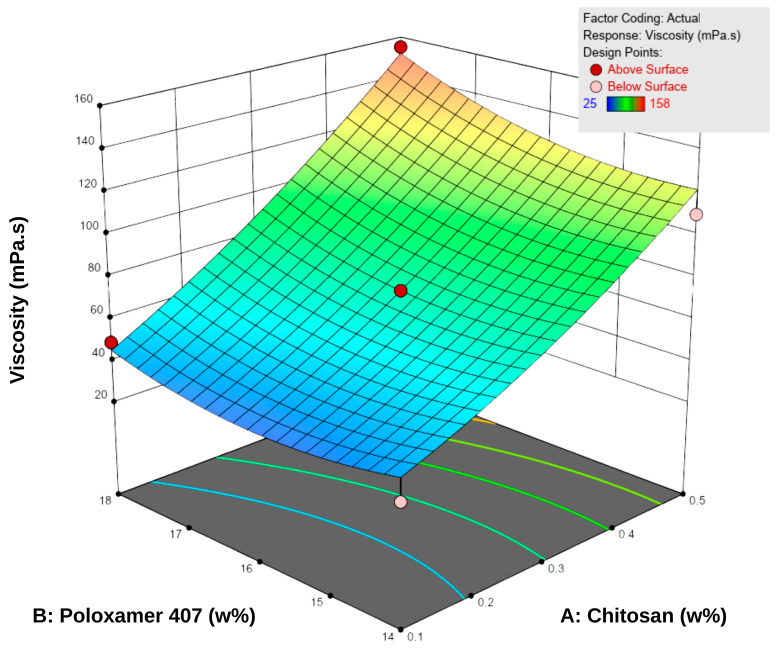
Three-dimensional response surface plot showing the effect of chitosan (**A**) and Poloxamer 407 (**B**) concentrations on the viscosity of the hydrogels. Increasing the concentration of both polymers resulted in a progressive rise in viscosity, as reflected by the surface gradient and contour lines.

Viscosity measurements were performed at 5 °C, as the formulations were intended to be stored under refrigerated conditions. The viscosity values of the spray gels ranged between 22.0 ± 1.0 and 157.7 ± 3.5 mPa·s (Table 5). The results showed that increasing the concentrations of both P407 and chitosan led to a significant increase in viscosity. According to the RSM analysis, both factors positively influenced viscosity (Figure 2). Examination of the response surface contour plots revealed that viscosity increased more markedly with higher chitosan concentrations, while the increase in P407 concentration supported this effect to a lesser extent. This difference is attributable to the different working ranges of the two polymers: chitosan concentration varied from approximately 0.1–0.5% (a nearly five-fold increase), whereas P407 concentration changed only between 14 and 18% (around a 30% increase). Overall, the interaction between the two factors was synergistic, demonstrating a positive combined effect on viscosity.

This behavior can be explained by the enhanced entanglement of chitosan macromolecular chains at higher concentrations and by the reinforcement of the three-dimensional network structure through the increased micelle density of P407 [18,40]. Additionally, micellar junction zones formed between chitosan and poloxamer may have contributed secondarily to the increase in viscosity [18]. Nevertheless, the viscosity values obtained remained within the acceptable range for spray-gel systems reported in the literature, and the formulations were considered suitable in terms of sprayability [10,41,42].

#### 3.1.2. Sprayability

**Figure 3:**
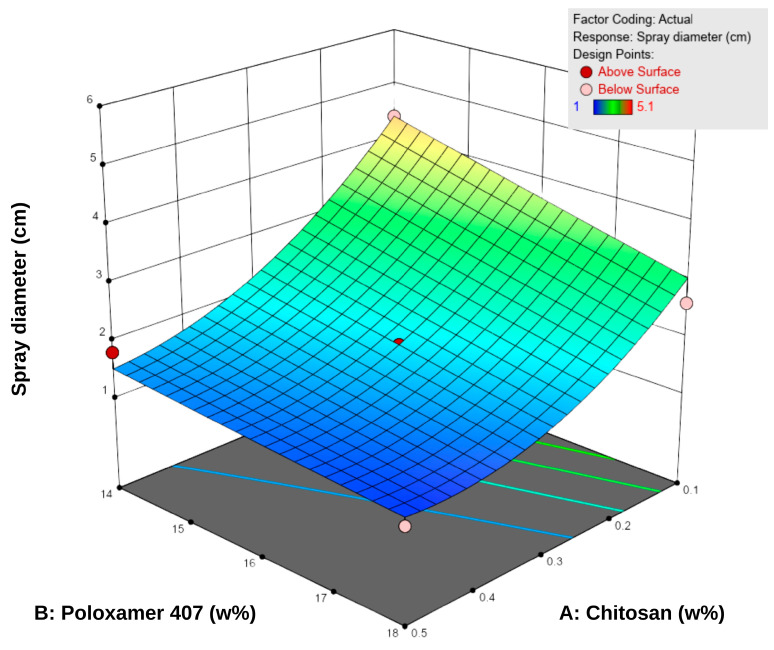
Three-dimensional response surface plot showing the effect of chitosan (**A**) and Poloxamer 407 (**B**) concentrations on the sprayability of the hydrogels. Higher polymer concentrations were associated with reduced sprayability, as reflected in the surface response trend.

The spray diameter values of the formulations ranged from 1.0 to 5.1 cm (Table 5). According to the RSM analysis, both chitosan and P407 negatively influenced the spray diameter (Figure 3). An increase in P407 concentration led to a significant reduction in spray area, and higher chitosan concentrations partially reinforced this effect. This observation can be attributed to the elevated polymer density, which elevates viscosity and limits droplet spreading during spraying [43]. Specifically, P407 reduces fluidity by increasing micelle formation at higher concentrations, whereas chitosan enhances the internal structural strength of the system by forming interchain hydrogen bonds [18,44,45,46].

The difference between low-viscosity formulations such as F1 (22 mPa·s, 5.1 cm) and high-viscosity formulations such as F8–F9 (155–158 mPa·s, 1.0–1.3 cm) clearly demonstrates the inverse relationship between viscosity and sprayability. Consistent with literature findings, low-viscosity systems produced wider spray areas, whereas high-viscosity systems exhibited narrower, more controlled spray profiles [12,47]. Overall, since the spray diameters were generally within the 2–5 cm range, the formulations were considered suitable for sprayability. However, optimal performance was achieved at medium viscosity levels (approximately 40–80 mPa·s), which provided a balance between effective spreading and skin adhesion.

#### 3.1.3. In Vitro Bioadhesion

**Figure 4:**
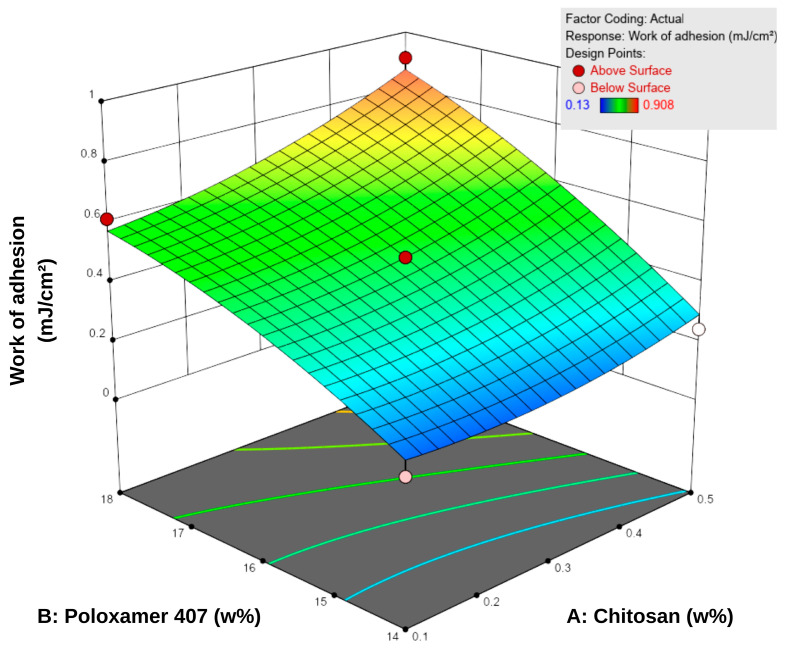
Three-dimensional response surface plot showing the effect of chitosan (**A**) and Poloxamer 407 (**B**) concentrations on the work of adhesion of the hydrogels. Increasing polymer concentrations led to higher adhesion values, as reflected in the response surface.

Bioadhesion is defined as the amount of work required to overcome the attractive forces that cause a formulation to adhere to a biological tissue or membrane in contact with its surface. This property is essential for enhancing therapeutic efficacy by prolonging the residence time of topical systems at the application site [45]. Based on the bioadhesion tests conducted using a texture analyzer, the in vitro work of adhesion of the formulations ranged from 0.130 to 0.908 mJ/cm^2^ (Table 5). According to the response surface contour plots (Figure 4), an increase in P407 concentration led to a more pronounced enhancement in bioadhesion, while increases in chitosan content provided a secondary yet consistent supportive effect. For instance, at a fixed chitosan level of 0.1%, increasing P407 concentration from 14% to 18% led to a 3–4-fold increase in bioadhesion (0.153–0.616 mJ/cm^2^). Conversely, increasing chitosan concentration while keeping P407 constant resulted in a more moderate increase of approximately 1.5–2-fold.

These findings indicate that P407 is the dominant factor governing the bioadhesive behavior of the system within the tested concentration range. This is attributed to increased micelle density and enhanced polymeric entanglement at higher P407 levels, which reinforce the compactness of the gel matrix and increase resistance to detachment [48]. Although chitosan is inherently bioadhesive, its contribution at lower concentrations was relatively limited compared to P407.

In hydrogel systems, a positive correlation between viscosity and bioadhesion is widely reported [32,49]. This relationship is primarily associated with increased polymer density, which enhances interchain hydrogen bonding and other secondary interactions, thereby improving structural cohesiveness and surface adhesion [50]. In this study, such a relationship was observed when one polymer concentration was held constant while the other was increased. Overall, the results demonstrate that increasing the total polymer concentration enhances bioadhesion; however, the magnitude of this effect depends on the relative proportions of P407 and chitosan.

A comparison between formulations F6 (0.3% chitosan and 18.83% P407) and F8 (0.5% chitosan and 18% P407) further illustrates the critical role of polymer ratios. In F6, the high P407 content increased gel network density and viscosity (98.0 mPa·s), resulting in moderate bioadhesion (0.667 mJ/cm^2^). In contrast, formulation F8 exhibited markedly higher viscosity and bioadhesion values (155.0 mPa·s; 0.908 mJ/cm^2^) due to increased chitosan content. This suggests that chitosan reinforces the poloxamer-based network by forming additional hydrogen bonds and electrostatic interactions, thereby enhancing surface adhesion. Therefore, an appropriate balance between P407 and chitosan concentrations yields synergistic interpolymer interactions and optimizes bioadhesion.

### 3.2. Determination of Lead Formulation by Desirability Approach

Two optimization strategies were applied using the software’s desirability function to balance the critical quality attributes. In the first model, the concentrations of chitosan and P407 were adjusted to maximize bioadhesion and spray diameter while minimizing viscosity. In the second model, similar response targets were set; however, the chitosan concentration was restricted to remain within its experimental range. Each approach yielded a single optimized composition, designated as S1 and S2, respectively.

**Figure 5:**
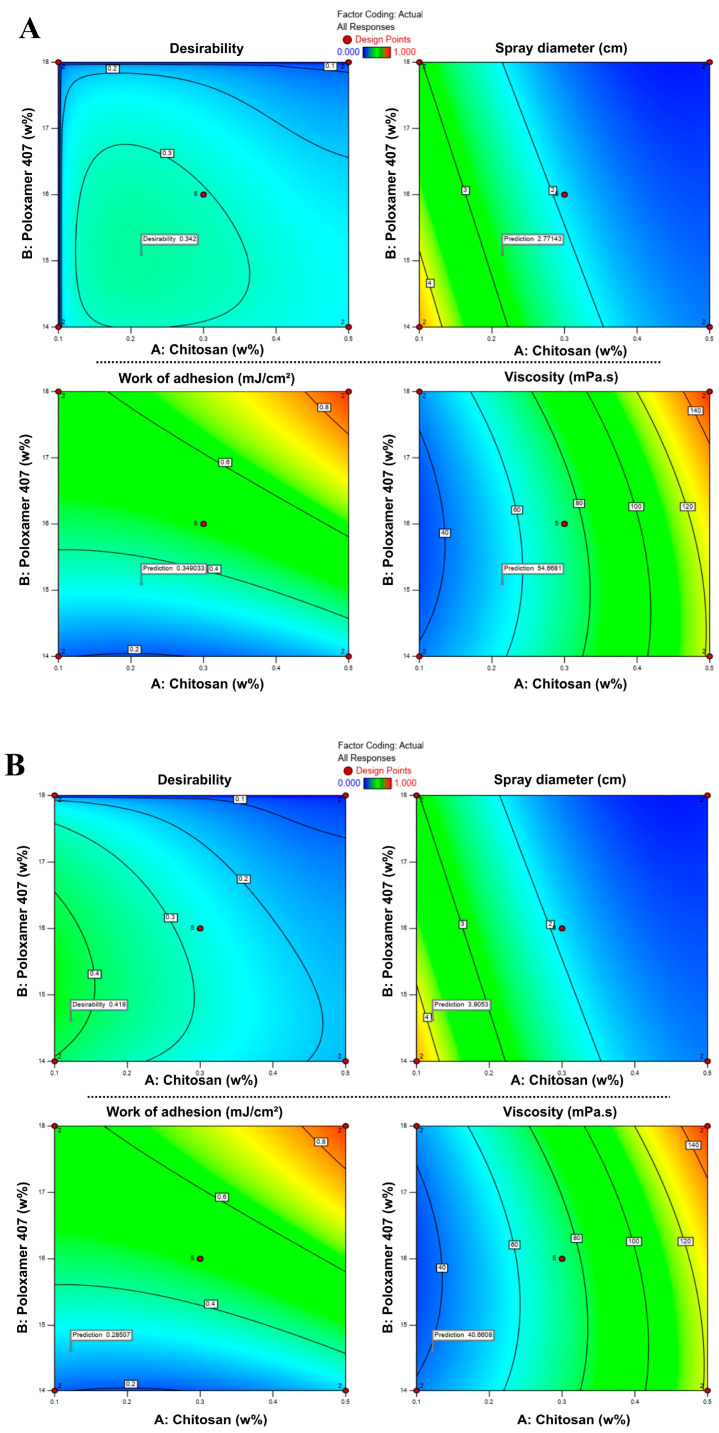
Response surface contour plots showing the effects of chitosan and P407 concentrations on desirability, spray diameter, viscosity, and bioadhesion in formulations S1 (**A**) and S2 (**B**). Color transitions represent changes in the response values, where blue indicates lower and red indicates higher values.

In RSM-based optimization, the combination that best satisfies the predefined criteria is represented by a desirability score ranging from 0 to 1 [51,52]. As shown in Figure 5, desirability values varied with the concentrations of chitosan and P407, and the optimum points were located within specific regions of the design space. In the first approach (S1), the model suggested an optimal formulation containing 0.214% chitosan and 15.10% P407, with a desirability score of 0.424. In the second approach (S2), the optimal composition was determined as 0.122% chitosan and 14.62% P407, with a desirability score of 0.543. These two optimized drug-loaded formulations, hereafter denoted as S1 and S2, and their compositions are summarized in Table 6.

Although the desirability values (0.424 and 0.543) were moderate rather than high, this did not indicate poor model performance. Instead, it reflected the antagonistic relationship among the responses: increasing bioadhesion was desirable; however, higher viscosity, which supports bioadhesion, simultaneously reduced spray diameter, making it impossible to maximize all targets. Therefore, the model identifies a rational compromise between these opposing responses. In pharmaceutical formulation studies with multiple objective functions, desirability scores in the range of 0.4–0.6 are widely reported as acceptable, representing a practical balance between conflicting quality attributes [51,52,53,54].

### 3.3. Characterization of Lead Hydrogels

#### 3.3.1. Viscosity, Sprayability, and In Vitro Bioadhesion

The model-predicted and experimental values for the two optimized formulations (S1 and S2), determined by the RSM analysis, are presented in Table 6. As expected, formulation S1—with higher chitosan and P407 contents—was predicted to exhibit higher viscosity and bioadhesion but a smaller spray diameter. The experimental outcomes for both formulations fell within the 95% prediction interval, confirming the model’s high predictive accuracy and strong alignment with experimental findings.

The experimental results showed that S1 (0.214% chitosan, 15.10% P407) had higher viscosity (*p* < 0.05) and bioadhesion (*p* > 0.05) but a smaller spray diameter (*p* < 0.05) compared to S2. Conversely, S2 (0.122% chitosan, 14.62% P407) demonstrated a more fluid structure, characterized by a larger spray diameter and lower viscosity. These results suggest that P407 concentration primarily influences the system’s flow resistance, whereas increasing chitosan concentration mainly enhances bioadhesion. The viscosity values of both formulations (42.7–58.7 mPa·s) were within the acceptable range reported for sprayable gel systems in the literature.

**Figure 6:**
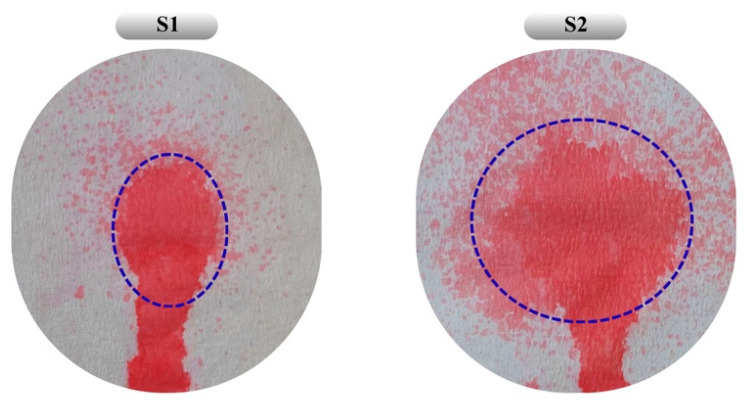
Distribution patterns obtained from the sprayability study of formulations S1 and S2. Blue dashed lines indicate the main spray area formed during the spraying process. The colored area below the dashed line represents the gravitational flow of the formulation along the vertically oriented surface. Under real application conditions, the system is expected to gel at body temperature, thereby ensuring retention at the application site.

The absence of a statistically significant difference in bioadhesion between the two formulations may be attributed to the relatively small increase in chitosan concentration and the minor difference in overall polymer content. Additionally, S2, with its lower chitosan content and wider spray diameter, appears more suitable for application over larger wound surfaces (Figure 6). As a result, both optimized formulations obtained by RSM and desirability-based optimization were evaluated as reliable systems achieving an appropriate balance of viscosity, sprayability, and bioadhesion.

The experimental values of viscosity, spray diameter, and bioadhesion for S1 and S2 were also compared with the model-predicted data (Table 6). The percent prediction errors for all responses ranged from 1.15% to 7.57%, remaining below the generally accepted 10% threshold [38,55]. These findings confirm the validity of the RSM model and indicate that the selected formulations are reliable outputs of the optimization process.

#### 3.3.2. pH

In topical wound care, the pH of drug delivery systems should minimize the risk of irritation or inflammatory reactions and be conducive to the wound-healing process. The pH value of healthy skin is slightly acidic and generally falls within the range of pH 4–6 [56,57]. However, pH dynamics follow a different course during wound healing. The literature reports that during the healing process of acute wounds, pH remains acidic during the inflammatory phase, rises slightly during the granulation phase, and returns to the pH range of 4–6 during the re-epithelialization phase. In contrast, chronic wounds generally exhibit a more alkaline pH (approximately 7–8), which is associated with a pathological environment that disrupts the healing process [58]. The pH of the formulations we developed (~4.5) is consistent with the physiologically accepted acidic range for acute wound healing and aligns well with the intended use of the designed product.

#### 3.3.3. Gelation Temperature (T_G_)

**Figure 7:**
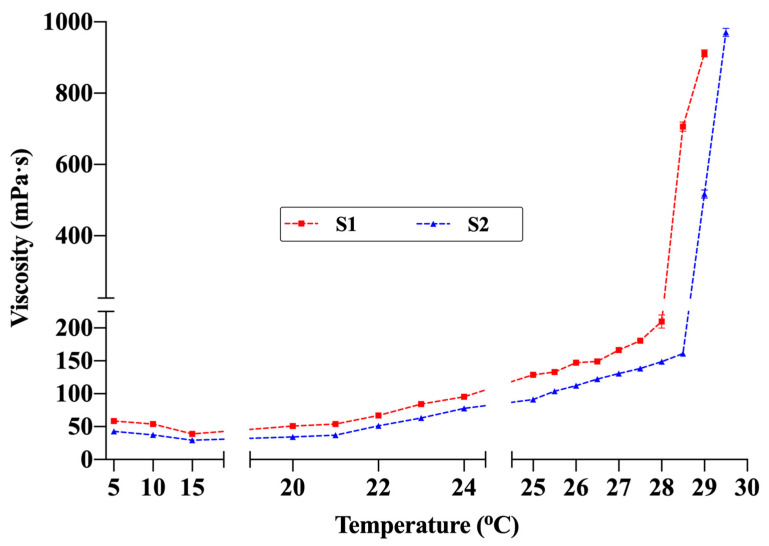
Viscosity changes in formulations S1 and S2 with temperature (Brookfield DV2T viscometer, SC21 spindle, 50 rpm). The maximum viscosity value that the device can measure under these conditions is 1000 mPa·s (n = 3).

The T_G_ values of the hydrogel formulations were determined as 28.7 ± 0.6 °C for S1 and 29.3 ± 0.3 °C for S2 (Table 7). Examination of the temperature-dependent viscosity profiles (Figure 7) revealed that viscosity remained low and stable between 5 °C and 20 °C. Maintaining fluidity within this range provides a significant advantage for spray application. The fact that the viscosity did not reach levels that would hinder spraying even at room temperature (≈22–25 °C) indicates that the formulations can maintain sprayability under typical use conditions. Viscosity began to increase gradually around 24 °C and rose sharply between 28 °C and 30 °C, marking the sol–gel transition. Under the measurement conditions (SC21 spindle, 50 rpm), the maximum measurable viscosity was 1000 mPa·s; thus, the final measurement was recorded at 29 °C for S1 and 29.5 °C for S2. These rheological findings, supported by visual observation, confirmed complete gelation. The slightly lower T_G_ of S1 compared with S2 is attributed to its higher concentrations of chitosan and P407. Accordingly, the temperature-dependent viscosity profiles showed excellent agreement with the T_G_ values determined by the conventional method. Additionally, comparative studies evaluating the effect of the active ingredients on T_G_ showed that drug-free formulations had lower T_G_ values (S1_blank_: 27.5 ± 0.5 °C; S2_blank_: 28.6 ± 0.9 °C). This finding suggests that lidocaine and allantoin slightly shift the gelation behavior upward by altering polymer–solvent interactions. These observations are consistent with previous studies on thermosensitive hydrogels, which have similarly reported shifts in gelation behavior upward when high concentrations of active ingredients were incorporated in dissolved form [10,41].

The obtained T_G_ values fall within the physiological range suitable for thermosensitive systems that undergo sol–gel transition at skin temperature. The primary driver of this transition is P407. Increasing the concentration of P407 promotes micelle formation and aggregation, leading to the development of a three-dimensional gel network. At low temperatures, the hydrophobic poly(propylene oxide) chains of P407 interact with water molecules via hydrogen bonding, thereby remaining dispersed. As the temperature rises, these hydrogen bonds weaken, hydrophobic interactions become dominant, and micelles move closer to one another, forming an ordered cubic or hexagonal structure characteristic of the gel phase [44,59]. Beyond this specific mechanism, a comprehensive review emphasizes that temperature-induced sol–gel transitions in polymer systems are generally governed by the balance of enthalpic and entropic effects within the polymer–solvent mixture. These transitions arise from temperature-dependent interactions of functional groups or macromolecular segments with each other or with surrounding water molecules, which collectively drive structural reorganization within the system [60].

Adjusting the T_G_ within an appropriate range is essential to ensure both easy sprayability and stable gel formation at body temperature. The literature generally considers T_G_ values below 35 °C as optimal for topical thermosensitive formulations [10,47,56]. This range enables rapid gelation and prolonged residence time at the application site while maintaining sprayability. Therefore, both formulations were confirmed to exhibit sprayable thermosensitive gel character for topical wound applications. Such temperature-responsive behavior ensures rapid stabilization on the wound surface after application and minimizes runoff or leakage from the target area, thereby enhancing local retention and therapeutic efficiency.

#### 3.3.4. Content Uniformity

The values obtained from content uniformity studies (Table 7) were within the pharmacopoeial limits (85–115%), confirming the repeatability of the preparation process and the homogeneity of the ingredients within the hydrogel.

### 3.4. Drug Release Studies

**Figure 8:**
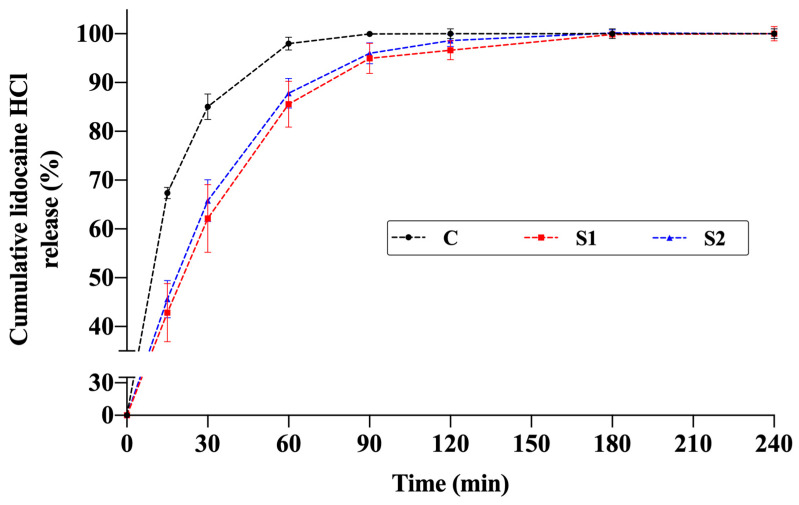
Cumulative in vitro release profiles of lidocaine from thermosensitive spray-gel formulations S1 and S2 and the aqueous control solution of lidocaine (C) (n = 4).

The release profiles of the thermosensitive spray gels (S1 and S2) were compared with an aqueous solution containing the same amount of active ingredient. In both formulations, lidocaine release increased markedly during the first 60–90 min and then reached a plateau, with cumulative release values stabilizing at approximately 95–100% around 120 min (Figure 8).

In this study, spectral overlap between allantoin and lidocaine at 211 nm resulted in insufficient specificity when using the dual-wavelength subtraction method with a UV–Vis spectrophotometer. Additional control experiments containing only allantoin demonstrated that allantoin release reached high values (79–93%) within the first 15 min. However, in formulations containing both lidocaine and allantoin, subtracting the absorbance of lidocaine from the total absorbance led to inaccurate and analytically unreliable quantification of allantoin, due to the strong overlap of their UV absorption bands in the 200–220 nm region. This spectral interference prevented the two actives from being distinguished using UV–Vis spectroscopy, making it impossible to determine an independent release profile for allantoin under the current analytical conditions. Therefore, release profiles were evaluated exclusively based on lidocaine. It is anticipated that future studies will benefit from chromatographic techniques such as HPLC-UV or LC–MS for the simultaneous and precise quantification of both active substances.

For the lidocaine control solution, the highest release rate was observed in the early phase due to the absence of a polymeric gel structure. In formulation S2, the lower viscosity resulting from lower P407 and chitosan concentrations facilitated faster diffusion, leading to higher release within the first 60 min compared to formulation S1. In formulation S1, the higher total polymer content formed a denser network, partially limiting early-phase diffusion but not affecting the final cumulative release, which reached values similar to the control at 120 min.

These findings indicate that although the semi-gel structure formed by the poloxamer–chitosan network partially retarded drug diffusion during the initial stage, it did not hinder the total drug release. Both formulations, S1 and S2, exhibited rapid release characteristics within a short period. However, it should be emphasized that this behavior is associated with the in vitro experimental conditions, particularly the dialysis membrane method and sink conditions with a large aqueous volume, and should not be directly interpreted as evidence of rapid analgesic onset under in vivo conditions.

A similar release behavior has been reported in the literature. In thermosensitive gels containing 20–30% P407, lidocaine hydrochloride release was prolonged and completed within 6 h; approximately 60% of the drug was released within the first 2 h. At higher polymer concentrations, the increased viscosity significantly slowed drug diffusion through the gel matrix [61]. In another study, microemulsion-based gel formulations containing Carbopol showed approximately 60% lidocaine release within the first 2 h, with viscosity values around 700–800 mPa·s [62]. Likewise, dual-responsive lidocaine in situ gel systems formulated using P407 and Gelrite^®^ for pain reduction during intrauterine device insertion exhibited 65–73% release within 2 h, whereas the aqueous control solution showed 100% release within 60 min [63]. These studies demonstrate that P407-based formulations can still provide rapid drug release under in vitro conditions, even with relatively high viscosity.

The release profiles obtained in the present study are generally consistent with these previous findings. However, the shorter release period (~2 h) observed in this work can be attributed to the lower P407 concentration and, consequently, lower viscosity. Although increased viscosity slowed early-phase diffusion, it did not limit total release. The rapid release observed can also be associated with the high aqueous solubility of lidocaine hydrochloride [64] and the ease of dispersion of poloxamer and chitosan glutamate salt in water [32,65].

Model-independent similarity factor (f_2_) analysis further supported these results. According to the established criterion, f_2_ ≥ 50 indicates similar profiles, while f_2_ < 50 indicates dissimilarity [10]. Comparison between the control solution and the gel formulations showed dissimilar profiles: C–S1: 44.53 and C–S2: 47.88. This verifies that the polymeric network alters release kinetics in the early phase. Conversely, the release profiles of S1 and S2 were similar to each other (f_2_ = 82.75), indicating that the two gel formulations demonstrated consistent release behavior while being significantly different from the control solution. This may imply a more controlled yet rapid release behavior suitable for topical applications.

### 3.5. Cytotoxicity Studies

**Figure 9:**
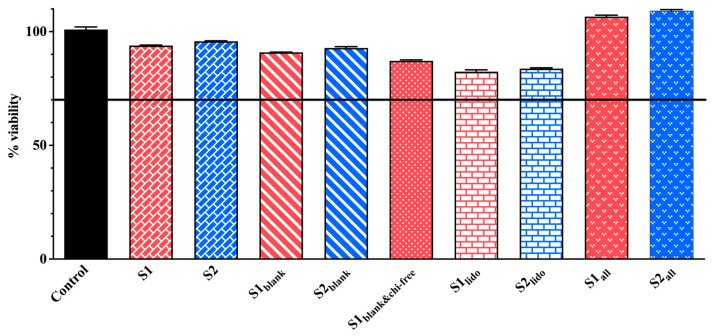
Cell viability of HaCaT keratinocytes following 24 h exposure to different formulations as determined by the MTT assay (n = 3). In these formulations, S1 and S2 represent drug-loaded systems containing both lidocaine and allantoin within a chitosan–P407 thermosensitive matrix. The variants S1_lido_ and S2_lido_ contain only lidocaine, whereas S1_all_ and S2_all_ contain only allantoin. S1_blank_ and S2_blank_ denote drug-free formulations composed solely of the chitosan-P407 matrix without active ingredients, while S1_blank&chi-free_ corresponds to a drug-free and chitosan-free formulation consisting exclusively of the P407 gel.

The MTT assays conducted on HaCaT keratinocytes demonstrated comparable cell viability across the formulations tested. All results are presented in Figure 9. Despite slight variations in chitosan and P407 levels, all S1 and S2 formulation pairs showed comparable cell viability, with no significant differences detected (*p* > 0.05). The formulations containing only allantoin (S1_all_ and S2_all_) exhibited viability rates of 106.58% and 109.15%, respectively, indicating a significant proliferative effect compared to the control. This finding aligns with previous reports indicating that allantoin promotes keratinocyte proliferation and supports epidermal regeneration [23,24,66].

Formulations containing both allantoin and lidocaine (S1 and S2) maintained high levels of cell viability, with measured values of 93.89% and 95.74%, respectively. Published studies support that lidocaine, at non-cytotoxic concentrations, does not compromise keratinocyte survival [67,68]. These observations are consistent with the maintained viability observed in our lidocaine-containing formulations. In the blank formulations, cell viability remained relatively high (S1_blank_: 90.88%, S2_blank_: 92.83%), whereas the formulation lacking chitosan glutamate (S1_blank&chi-free_) displayed slightly lower viability (87.15%). The slightly reduced viability observed in the chitosan-free formulation may further indicate chitosan’s contribution to cellular compatibility. Several studies have demonstrated that chitosan and its derivatives, including chitosan glutamate, are biocompatible materials that enhance keratinocyte adhesion, proliferation, and migration, thereby contributing to epithelial repair processes [32,69,70]. In the lidocaine-only groups (S1_lido_: 82.41% and S2_lido_: 83.76%), viability values were lower than in other blank or active ingredient-loaded formulations. However, both groups retained cell survival rates above 80%, indicating that lidocaine was not cytotoxic at the tested concentration. Although lidocaine has been reported to cause significant decreases in viability in HaCat cells at increasing concentrations, it was biocompatible at appropriate concentrations [71]. In one study, Qiao et al. demonstrated that microemulsion-based gel formulations containing 5% lidocaine maintained 80% cell viability in rat skin keratinocytes for 14 days [72]. Formulations containing allantoin demonstrated enhanced cell viability, while those containing both active ingredients maintained healthy cell populations comparable to the control. The combination of allantoin’s proliferative effect, chitosan glutamate’s biocompatibility, and lidocaine’s low cytotoxicity likely contributed to the favorable viability profiles observed. These findings collectively support that none of the tested active ingredients—alone or in combination—exerted adverse effects on HaCaT keratinocytes, and that allantoin-containing formulations may even stimulate proliferative activity.

### 3.6. Wound-Healing Activity of Formulations on HaCaT Keratinocytes

**Figure 10:**
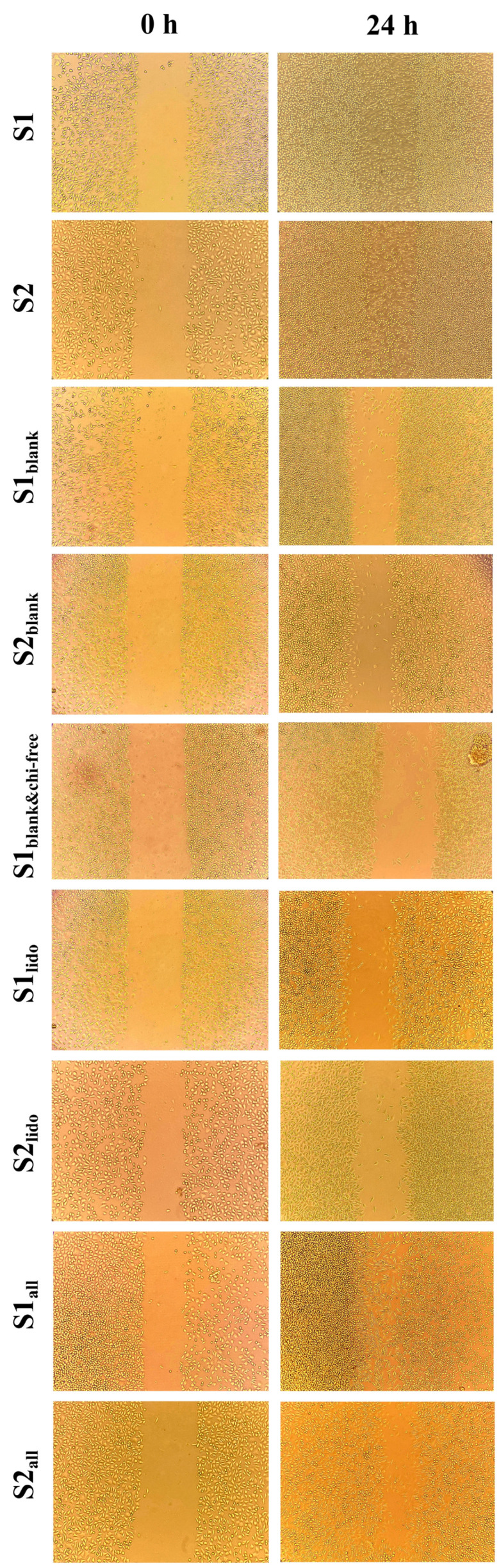
Evaluation of the effects of different formulations on cell migration in HaCaT cells using a scratch assay. Images are shown at 0 and 24 h following application of the formulations to cells.

The wound-healing potential of the formulations was evaluated using an in vitro scratch assay on HaCaT keratinocytes at 0 and 24 h (Figure 10). Scratch assay results were assessed qualitatively rather than quantitatively. In this experiment, minor variations in initial scratch geometry and the irregular advance of cells from the wound edges made precise boundary detection challenging, and therefore, qualitative interpretation was considered more reliable for comparing overall migration trends. Although there were slight differences in initial cell density and scratch width between formulations, the narrowing of the scratch line after 24 h in each group was sufficient to understand the general trend in HaCaT cell migration. The images qualitatively demonstrate which formulations promoted cell migration more, and these observations provide a migration profile consistent with other cellular data. After 24 h, the wound area in the S1 and S2 formulations, which contained both allantoin and lidocaine, showed complete wound closure, demonstrating the strongest regenerative response among all the tested formulations. Formulations containing only allantoin (S1_all_ and S2_all_) also enhanced wound closure, showing a marked increase in cellular migration compared to the lidocaine-only groups. By comparison, the lidocaine-only formulations (S1_lido_ and S2_lido_) exhibited slower closure rates, with partial recovery of the wound area at 24 h. This finding suggests that lidocaine alone provides limited regenerative support under the experimental conditions, as its primary contribution is anti-inflammatory rather than proliferative. Interestingly, the blank formulations containing chitosan glutamate (S1_blank_ and S2_blank_) promoted moderate cell migration, whereas the formulation lacking chitosan glutamate (S1_blank&chi-free_) resulted in noticeably slower wound closure. This specific group was designed to evaluate the role of chitosan glutamate in wound healing. This observation implies that chitosan glutamate contributes to keratinocyte migration and may enhance the bioactivity of the topical matrix. Chitosan and its derivatives, which are non-toxic, biocompatible, and biodegradable, have been reported to possess significant clinical potential in wound healing, particularly in dosage forms such as gels, films/patches, sponges, and scaffolds [73]. The superior performance of the S1 and S2 compared with the allantoin-only groups suggests a synergistic interaction between allantoin and lidocaine, where allantoin primarily accelerates epithelial regeneration, while lidocaine mitigates inflammatory stress, thereby creating a favorable microenvironment for cell proliferation and migration.

The wound-closure effects observed in this study are in agreement with previously reported data on the regenerative and anti-inflammatory properties of the individual components. Allantoin has been extensively described as a potent wound-healing agent that enhances fibroblast proliferation, extracellular matrix formation, and epithelial migration [24]. Lidocaine, on the other hand, exhibits dose-dependent effects: it is non-cytotoxic at low concentrations and exerts anti-inflammatory activity by suppressing TNF-α and NF-κB signaling, which may indirectly facilitate the wound-healing process [22]. Furthermore, chitosan has been shown to support keratinocyte proliferation and modulate inflammatory cytokines such as TNF-α, enhancing overall tissue repair [74,75]. The enhanced wound healing observed in the combined formulations can be attributed to a synergistic mechanism involving both active compounds. Allantoin primarily contributes to wound repair by stimulating cellular regeneration and by promoting epithelialization, whereas lidocaine complements this effect by attenuating inflammatory signaling. In particular, lidocaine-mediated downregulation of pro-inflammatory cytokines such as TNF-α reduces cellular stress and creates a more favorable microenvironment for keratinocyte proliferation and migration. The synergy between these actions results in a faster and more coordinated wound-repair process. Supported by chitosan, these interactions create a microenvironment that accelerates keratinocyte proliferation and migration, resulting in complete wound closure within 24 h.

In summary, formulations containing both allantoin and lidocaine exhibited the strongest wound-healing effect, exceeding that observed with either compound alone. These findings suggest a synergistic mechanism in which allantoin drives tissue regeneration, while lidocaine modulates inflammation, ultimately promoting accelerated wound repair in HaCaT keratinocytes.

### 3.7. Effect of Formulations on TNF-α Expression Levels

**Figure 11:**
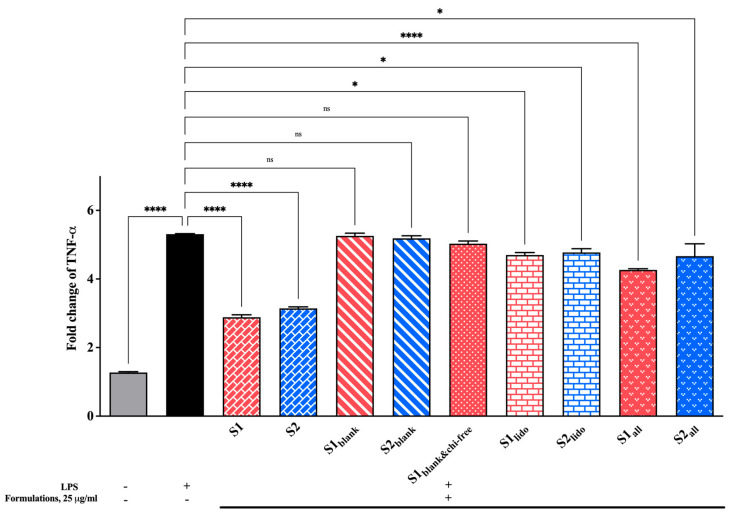
Fold change in TNF-α expression in HaCaT keratinocytes following 24 hr of treatment with different formulations (25 μg/mL). LPS stimulation significantly increased TNF-α expression, while allantoin- and lidocaine-containing formulations (S1 and S2) markedly suppressed cytokine levels (* *p* < 0.05, **** *p* < 0.0001, ns = non-significant). Data are expressed as mean ± SD (n = 3).

Changes in TNF-α levels, a key pro-inflammatory cytokine in the inflammatory cascade, were examined to evaluate the formulations’ anti-inflammatory potential. All findings are presented in Figure 11. The LPS-stimulated control group showed a pronounced increase in TNF-α expression compared to the unstimulated cells, confirming the successful induction of an inflammatory response. Treatment with the formulations containing both allantoin and lidocaine (S1 and S2) markedly reduced TNF-α levels, significantly suppressing them relative to the LPS control (**** *p* < 0.0001). Among the groups, the allantoin-only formulations (S1_all_ and S2_all_) also demonstrated a moderate but consistent reduction in TNF-α expression, whereas the lidocaine-only groups (S1_lido_ and S2_lido_) produced slightly less pronounced effects. Blank formulations containing chitosan glutamate (S1_blank_ and S2_blank_) and the formulation lacking chitosan glutamate (S1_blank&chi-free_) did not provide a statistically significant change in TNF-α expression compared to the LPS control (p > 0.05). As a result, these findings indicate that formulations containing allantoin and lidocaine effectively attenuate the TNF-α–mediated inflammatory response. The suppression of TNF-α expression can be attributed to the combined and complementary effects of the active ingredients.

Chitosan derivatives have been shown to reduce the release of inflammatory cytokines, including TNF-α, in keratinocyte and wound-healing models. For example, chitosan-based nanomaterials significantly downregulated TNF-α and IL-6 expression, while promoting epithelial regeneration [74]. Similarly, lidocaine has demonstrated anti-inflammatory activity by inhibiting cytokine secretion, including TNF-α, in both keratinocytes and immune cell models [76]. A previous study reported that lidocaine exhibits no cytotoxic effects at low concentrations and concurrently reduces inflammatory cytokines—particularly TNF-α—indicating that these properties may contribute to enhanced wound-healing outcomes [22]. Additionally, allantoin—widely recognized for its wound-healing and keratinocyte-regenerative properties—can reduce local inflammation and accelerate tissue repair when combined with biopolymers such as chitosan and collagen [28]. These data suggest that the observed decrease in TNF-α expression in allantoin- and lidocaine-loaded formulations results from both direct anti-inflammatory actions of the active compounds and the supportive effects of the chitosan-based delivery matrix. The combined use of allantoin and lidocaine appears to maintain cellular integrity while modulating inflammatory signaling.

Overall, the formulations containing allantoin, lidocaine, and chitosan glutamate effectively mitigated LPS-induced TNF-α overexpression, reflecting a promising anti-inflammatory potential suitable for topical wound-healing applications.

## 4. Conclusions

In this study, a thermosensitive and sprayable hydrogel system containing lidocaine and allantoin was successfully developed, optimized, and evaluated through in vitro characterization. Using RSM, the effects of chitosan and P407 concentrations on viscosity, sprayability, and bioadhesion were statistically assessed, leading to the identification of two optimized formulations (S1 and S2). Both formulations could be easily sprayed at room temperature due to their low viscosity and exhibited a rapid sol–gel transition around 28–30 °C, forming a stable gel layer on the skin surface. Despite the presence of a gel matrix, lidocaine was rapidly released under sink conditions, reaching almost complete diffusion within 120 min. In addition, cytotoxicity, cell migration, and inflammation marker analyses revealed that both formulations exhibited high biocompatibility with keratinocytes and that the combined use of lidocaine and allantoin positively influenced cellular healing-related processes.

These findings suggest that chitosan–poloxamer-based spray gels, with their non-contact application, appropriate viscosity range, rapid gelation, bioadhesive properties, and favorable in vitro effects on cell viability, migration, and inflammation, offer a promising alternative to conventional semi-solid formulations such as creams and ointments. In addition, combining lidocaine’s rapid analgesic effect with allantoin’s healing activity in a single formulation highlights the dual therapeutic potential of the system from a clinical perspective. However, the current study is limited to in vitro investigations, and the inability to confirm allantoin release using a specific analytical method, such as HPLC, is a notable methodological limitation. In future stages, simultaneous quantification of both active substances via HPLC-UV or LC–MS and in vivo wound-healing experiments are planned. As a result, these findings provide a strong scientific basis for the potential clinical applications of these thermosensitive spray hydrogels in wound management.

## Figures and Tables

**Table 1:** Independent variables and their corresponding level for lidocaine–allantoin spray gel.

Independent Variable	Symbol	Code Levels				
		−α	−1	0	+1	+α
**Chitosan**	X1	0.017	0.1	0.3	0.5	0.583
**P407**	X2	13.17	14	16	18	18.83

**Table 2:** Experimental design matrix generated by the software.

Run	Formulation Code	Factor 1 (A) (Chitosan, *w*%)	Factor 2 (B) (P407, *w*%)
**1**	F5	0.3	16
**2**	F5	0.3	16
**3**	F4	0.3	13.17
**4**	F2	0.1	14
**5**	F5	0.3	16
**6**	F1	0.017	16
**7**	F7	0.5	14
**8**	F7	0.5	14
**9**	F2	0.1	14
**10**	F8	0.5	18
**11**	F4	0.3	13.17
**12**	F6	0.3	18.83
**13**	F3	0.1	18
**14**	F8	0.5	18
**15**	F5	0.3	16
**16**	F1	0.017	16
**17**	F5	0.3	16
**18**	F6	0.3	18.83
**19**	F9	0.58	16
**20**	F3	0.1	18
**21**	F9	0.58	16

**Table 3:** Statistical model summary and adequacy parameters for response variables in RSM optimization.

Response	Transform	Model	R^2^	Adjusted R^2^	Predicted R^2^	Adeq Precision	F-Value	*p*-Value
Viscosity	No transform	Quadratic	0.9712	0.9616	0.9301	28.7423	101.29	<0.0001
Spray diameter	No transform	Quadratic	0.9624	0.9499	0.9205	24.2616	76.80	<0.0001
Work of adhesion	No transform	Quadratic	0.9704	0.9605	0.9374	31.6013	98.37	<0.0001

**Table 4:** ANOVA table for the response surface quadratic model.

Source	Sum of Square	Df	Mean Square	F-Value	*p*-Value
Viscosity					
Model	37334.54	5	7466.91	101.29	<0.0001
A-Chitosan	34799.11	1	34799.11	472.07	<0.0001
B-P407	1130.39	1	1130.39	15.33	0.0014
AB	364.50	1	364.50	4.94	0.0420
A^2^	479.07	1	479.07	6.50	0.0222
B^2^	895.20	1	895.20	12.14	0.0033
CV (%)	9.95				
Spray diameter					
Model	30.76	5	6.15	76.80	<0.0001
A-Chitosan	22.92	1	22.92	286.09	<0.0001
B-P407	2.97	1	2.97	37.12	<0.0001
AB	0.5000	1	0.5000	6.24	0.0246
A^2^	3.87	1	3.87	48.35	<0.0001
B^2^	0.0016	1	0.0016	0.0201	0.8890
CV (%)	11.91				
Work of adhesion					
Model	1.12	5	0.2246	98.37	<0.0001
A-Chitosan	0.1446	1	0.1446	63.35	<0.0001
B-P407	0.8897	1	0.8897	389.68	<0.0001
AB	0.0202	1	0.0202	8.85	0.0094
A^2^	0.0210	1	0.0210	9.18	0.0084
B^2^	0.0232	1	0.0232	10.17	0.0061
CV (%)	9.83				

Df: Degrees of freedom, CV: Coefficient of variation.

**Table 5:** Results for three key response parameters (spray pattern diameter, viscosity, and work of bioadhesion) of the drug-loaded formulations (F1–F9) designed according to the RSM model.

Formulation Code	Independent Variables		Response Values		
	Chitosan (*w*%)	P407 (*w*%)	Viscosity at 5 °C (mPa·s ± SD)	Spray Diameter (cm ± SD)	Work of Bioadhesion (mJ/cm^2^ ± SD)
**F1**	0.017	16	22.0± 1.0	5.1 ± 0.1	0.450 ± 0.048
**F2**	0.1	14	30.7 ± 2.5	4.4 ± 0.2	0.153 ± 0.051
**F3**	0.1	18	49.3 ± 3.1	2.6 ± 0.2	0.616 ± 0.110
**F4**	0.3	13.17	56.7 ± 1.5	2.3 ± 0.1	0.130 ± 0.037
**F5**	0.3	16	73.7 ± 4.5	1.9 ± 0.0	0.488 ± 0.048
**F6**	0.3	18.83	98.0 ± 6.1	1.7 ± 0.0	0.667 ± 0.094
**F7**	0.5	14	110.3 ± 2.9	1.8 ± 0.1	0.244 ± 0.045
**F8**	0.5	18	155.0 ± 3.0	1.0 ± 0.1	0.908 ± 0.059
**F9**	0.58	16	157.7 ± 3.5	1.3 ± 0.1	0.717 ± 0.178

**Table 6:** Model-predicted and experimentally observed viscosity, spray diameter, and bioadhesion values for optimized formulations S1 and S2. Predicted values represent the “Predicted Median” results generated by the RSM model, and observed values represent the experimental data measured as mean ± SD (*n* = 3 for sprayability and viscosity, *n* = 4 for bioadhesion) (* *p* < 0.05).

Formulation code	Chitosan (%)	P407 (%)	Spray Diameter		Work of Bioadhesion		Viscosity	
			Predicted Median (cm)	Observed (cm ± SD)	Predicted Median (mJ/cm^2^)	Observed (mJ/cm^2^ ± SD)	Predicted Median (mPa·s)	Observed (mPa·s ± SD)
S1	0.214	15.100	2.77	2.56 ± 0.10 ^*^	0.349	0.345 ± 0.079	54.7	58.7 ± 0.6 ^*^
S2	0.122	14.620	3.91	4.06 ± 0.10	0.285	0.294 ± 0.029	40.7	42.7 ± 1.2

**Table 7:** pH, T_G_, and content uniformity of optimized spray-gel formulations (S1 and S2) (n = 3).

Formulation Code	pH (± SD)	T_G_ (°C ± SD)	Content Uniformity (% ± SD)	
			Lidocaine Hydrochloride	Allantoin
**S1**	4.35 ± 0.05	28.7 ± 0.6	99.88 ± 0.39	98.43 ± 2.71
**S2**	4.52 ± 0.08	29.3 ± 0.3	100.16 ± 0.64	98.77 ± 2.13

## Data Availability

The original contributions presented in this study are included in the article. Further inquiries can be directed to the corresponding authors.

## Abbreviations

The following abbreviations are used in this manuscript:

ANOVA	One-way analysis of variance
C	Control solution
CV	Coefficient of variance
Df	Degrees of freedom
DMEM	Dulbecco’s Modified Eagle Medium
HaCaT	Human keratinocyte cell line
HPLC	High-pressure liquid chromatography
IL-6	Interleukin-6
LC-MS	Liquid chromatography–mass spectrometer
MTT	3-(4,5-dimethylthiazol-2-yl)-2,5-diphenyltetrazolium bromide
MWCO	Molecular weight cut-off
P407	Poloxamer 407
QbD	Quality by design
RSM	Response surface methodology
SD	Standard deviation
T_G_	Gelation temperature
TNF-α	Tumor necrosis factor-alpha
UV	Ultraviolet
